# Practical Notes on Diagnosis and Treatment

**Published:** 1907-10-19

**Authors:** 


### Practical Notes on Diagnosis and Treatment.

**Time Influence of Syphilis.**

It is seldom that any syphilitic process develops thirty years after the first onset of the malady, whether it be a direct or an indirect syphilitic process. —*Sir Wm. Gowers.*

**Chorea.**

In chronic cases of chorea it is good treatment to give cold douching down the spine, preceded by a hot bath. This method is not suitable for acute and severe cases.—*Dr. Herringham.*

**Hay Fever.**

The following may be painted inside the nose at the outset of an attack of hay fever: Menthol grs. xx., almond oil ʒij., carbolic acid m.x., cocaine hydrochloride grs. vj., zinc ointment ½ oz.

**Atropine in Graves' Disease.**

The action of atropine in Graves' disease is probably chiefly due to its lessening thyroid gland secretion, and is thus comparable to thyroidectomy. In using atropine in this disease the drug ought to be pressed to its physiological limit.—*Dr. Watson Williams.*

**Cancer of the Larynx.**

There is no part of the body in which cancer occurs which is more favourable for cure than inside the larynx, just as there is no part in which it is less favourable than when it has become extrinsic to the larynx. Hence, there is an urgent appeal for early diagnosis by laryngoscopic examination in cases of hoarseness even when this symptom exists in but a slight degree.—*Dr. St. Clair Thomson.*

**Treatment of Dysmenorrhœa.**

For genuine spasmodic dysmenorrhœa, in which it is not desirable to employ local treatment, guaiacum is the best drug. You may give the resin mixed with extract of malt or honey. Aletris cordial with chlorate of potassium will also sometimes prevent menstrual pain, whilst aspirin, antipyrin and phenacetin may be given to relieve the pain when it occurs.—*Dr. Herman.*

**Salicylic Ointment in Rheumatism.**

Salicylic acid in the form of an ointment is a very useful application to the painful joints of acute rheumatism. The formula advised is as follows: Salicylic acid, oil of turpentine, lanoline, of each ½ oz., lard 1½ oz. The ointment is to be spread on linen and applied to the joint; over this is arranged a layer of gutta-percha tissue, which is fixed in position by a flannel bandage. The ointment is also serviceable in gout and in chronic rheumatism.

**Morphine in Cardiac Disease.**

I am sure that in conditions of urgent cardiac distress, with restlessness and insomnia, we are far too fearful of morphine, and patients are allowed to die of sheer exhaustion for want of sleep. I fancy it can be given most safely in the form of a suppository, but, if given subcutaneously, it may be advantageously combined with strychnine. It is useless to give morphine if we have not the courage to give an efficient dose, and it is idle in advanced cardiac disease to put one's trust in hypnotics other than morphine.—*Dr. Raymond Crawford.*

**Morphine Poisoning.**

It should be remembered that in children an overdose of morphine may cause convulsions.

**The Pulse in Neurasthenia.**

In neurasthenia when the pulse tension is high the prognosis is better than when the tension is low.—*Sir Wm. Broadbent.*

**Dyspepsia.**

In atonic dyspepsia the following is a useful draught, taken half to one hour before meals: Sodium bicarbonate gr. xv. Tr. nucis vom. m. xv., Tr. calumbæ ʒss., Sp. ammon. arom. ʒss., Inf. auranti co. ad ℥j.

**Treatment of Dropsy.**

In obstinate cases of dropsy a pill containing calomel ½ gr., digitalis and squill, of each 1 grain, is often most successful. A dose should be given every three hours until diuresis, or free purgation, is established. In renal dropsies sparteine sulphate should not be forgotten. It may be given to adults in doses of ¼ grain, so that 2, or even 3, grains are taken in twenty-four hours.—*Dr. Tyson.*

**Syphilis of the Rectum.**

Syphilitic ulcer of the rectum should be treated by giving iodides internally, and applying a local mercurial. It is very much easier to apply an ointment than lotions to the rectum; therefore the best plan is to let the patient have some sort of bougie with which she cannot do herself any harm, smear it with a little blue ointment, and have it pushed up every night.—*Mr. Christopher Heath.*

**Olive-oil and Gall-stones.**

It has been observed in some cases that people who take a considerable quantity of olive oil with their food get rid of the obstruction of gall-stones more quickly and with less pain than others. It is said to be due to softening of the stone by the oil. Anyhow, the stone is got rid of. I have seen a few cases of that kind, and it is a remedy which one can quite safely try.—*Dr. Norman Moore.*

**Corneal Ulcer.**

Intractable corneal ulcer will sometimes yield to the following treatment: Cleanse the eye with warm sterilised boric acid lotion, then introduce a few drops of a weak cocaine solution, and after allowing a few minutes for anæsthesia to develop, touch the ulcer with a camel-hair pencil dipped in carbolic acid 15 grains, tannic acid 30 grains, glycerin 1 drachm. Repeat two or three times daily. With this application the use of atropine must be associated.

**Hydro-Pneumothorax in Phthisis.**

We have had many notable instances of the dangers of removing fluid in such cases. The compression of the tubercular lung seems to have a powerful effect in arresting the tubercular process; when the compression is relieved by taking away the fluid you frequently get an outburst of tubercular inflammation, and not infrequently of a miliary character. The indications for interference are when the dyspnœa is excessive or when the condition of the patient seems to indicate the absorption of pus.—*Dr. Habershon.*

